# Surveillance of Sarcoptic Mange in Iberian Ibexes (*Capra pyrenaica*) and Domestic Goats (*Capra hircus*) in Southern Spain

**DOI:** 10.3390/ani14081194

**Published:** 2024-04-16

**Authors:** Félix Gómez-Guillamón, Débora Jiménez-Martín, Debora Dellamaria, Antonio Arenas, Luca Rossi, Carlo V. Citterio, Leonor Camacho-Sillero, Barbara Moroni, David Cano-Terriza, Ignacio García-Bocanegra

**Affiliations:** 1 Programa de Vigilancia Epidemiológica de la Fauna Silvestre (PVE), Consejería de Sostenibilidad, Medio Ambiente y Economía Azul, Junta de Andalucía, 29002 Málaga, Spain; felixj.gomezguillamon@juntadeandalucia.es (F.G.-G.); leonorn.camacho@juntadeandalucia.es (L.C.-S.); 2 Departamento de Sanidad Animal, Grupo de Investigación en Sanidad Animal y Zoonosis (GISAZ), UIC Zoonosis y Enfermedades Emergentes ENZOEM, Universidad de Córdoba, 14014 Córdoba, Spain; debora.djm@gmail.com (D.J.-M.); arenas@uco.es (A.A.); nacho.garcia@uco.es (I.G.-B.); 3 Istituto Zooprofilattico Sperimentale delle Venezie, 35020 Legnaro, Italy; ddellamaria@izsvenezie.it (D.D.); ccitterio@izsvenezie.it (C.V.C.); 4 Department of Veterinary Sciences, University of Turin, 10095 Grugliasco, Italy; luca.rossi@unito.it; 5 Istituto Zooprofilattico Sperimentale del Piemonte, Liguria e Valle d’Aosta, via Bologna 148, 10154 Torino, Italy; barbara.moroni@izsto.it; 6 CIBERINFEC, ISCIII—CIBER de Enfermedades Infecciosas, Instituto de Salud Carlos III, 28029 Madrid, Spain

**Keywords:** Caprinae, domestic goat, mite, *Sarcoptes*, wild goat, monitoring

## Abstract

**Simple Summary:**

A serosurvey study was conducted in southern Spain to assess the exposure and spatial distribution of *Sarcoptes scabiei* in Iberian ibexes (*Capra pyrenaica*) and domestic goats (*Capra hircus*). The study included sera from 411 Iberian ibexes (157 with skin lesions compatible with sarcoptic mange and 254 that were clinically healthy), skin samples from 88 affected animals, and 392 serum samples from domestic goats, collected between 2015 and 2021. Antibodies against *S. scabiei* were found in 3.1% of the clinically healthy ibexes and 66.2% of those with compatible skin lesions. Mites were confirmed in 64.8% of the skin samples, and 86.0% of these mite-positive individuals had antibodies. Seropositive animals were detected in population nuclei with previous records of sarcoptic mange, but not in historically free population nuclei. The non-detection of antibodies against *S. scabiei* in the domestic goats suggests an independent epidemiological cycle of sarcoptic mange in Iberian ibex populations in the study area. Integrated surveillance programs and control strategies in wildlife and livestock are essential to mitigating the risk of *S. scabiei* circulation in Iberian ibex populations.

**Abstract:**

Sarcoptic mange is a highly contagious skin disease caused by *Sarcoptes scabiei*. Sera were collected from 411 Iberian ibexes, comprising 157 individuals with sarcoptic mange skin lesions and 254 clinically healthy animals, in 13 population nuclei across Andalusia (southern Spain) between 2015 and 2021. Skin samples from 88 of the 157 animals with mange-compatible lesions were also obtained. Moreover, 392 serum samples from domestic goats (*Capra hircus*) were collected in the same region and study period. Antibodies against *S. scabiei* were tested using an in-house indirect ELISA, while the presence of mites of *S. scabiei* was evaluated in the skin samples by potassium hydroxide digestion. Seropositivity was found in eight (3.1%) of the clinically healthy ibexes and in 104 (66.2%) of the animals with mange-compatible lesions. The presence of *S. scabiei* was confirmed in 57 (64.8%) out of the 88 skin samples analysed and anti-*S. scabiei* antibodies were found in 49 (86.0%) of these 57 mite-positive individuals. Seropositive animals were detected in population nuclei with previous records of sarcoptic mange, where *S. scabiei* mites were detected by potassium hydroxide digestion in individuals with sarcoptic mange-compatible external lesions. However, seropositivity was not observed in population nuclei that were historically free of this disease. None of the 392 domestic goats had antibodies against *S. scabiei*, suggesting an independent epidemiological cycle of sarcoptic mange in Iberian ibex populations in the study area, and a limited or null role of domestic goats in the transmission of the parasite to this wild species. Overall, our findings underscore the importance of maintaining and/or implementing integrated surveillance programs and control strategies in wildlife and livestock, to limit the risk of *S. scabiei* circulation in Iberian ibex populations.

## 1. Introduction

Sarcoptic mange, caused by the obligate burrowing mite *Sarcoptes scabiei*, is a highly contagious skin disease distributed worldwide [1,2]. This mite is considered one of the terrestrial ectoparasites with the widest host range, affecting both wildlife and domestic animals, and even humans [3]. In fact, *Sarcoptes scabiei* currently affects more than 100 mammalian species worldwide, posing an ongoing challenge to wildlife conservation and management in particular, as it has been associated with significant declines in local populations for decades [4].

Clinical manifestations of sarcoptic mange include intense pruritus, hair loss, scaling, and crusting or hyperkeratosis, among others [5]. Although the severity and clinical outcomes of sarcoptic mange can differ among species, populations, and individuals [6], it remains likely to be the most severe disease affecting wild Caprinae in Europe [7,8].

The Iberian ibex, or Iberian wild goat (*Capra pyrenaica*), is a distinctive endemic medium-sized wild ruminant emblematic of the Iberian Peninsula. Traditionally, four different subspecies of Iberian ibex have been described: *C. pyrenaica lusitanica,* which formerly inhabited northern Portugal and certain regions of northwestern Spain; *C. p. pyrenaica*, in the Pyrenees Mountains; *C. p. hispanica*, found in the southern and eastern regions of the Iberian Peninsula; and *C. p. victoriae*, distributed mainly in central areas of Spain [9]. At present, only two of the four subspecies originally described are not extinct (*C. p. hispanica* and *C. p. victoriae*) with a population of around 100,000 individuals that is expanding, or at least stable, in southern, central, and eastern regions of Spain, with smaller, localized populations also found in northern Portugal and southern France [10].

The Iberian ibex has been shown to be particularly sensitive to *S. scabiei* infection, which results in high rates of morbidity and mortality, especially in naïve populations [7]. In this regard, the first known epizootic outbreak of sarcoptic mange that affected this species caused an over 95% decline in an estimated population of almost 9500 individuals [11,12]. The introduction of infected domestic goats was the most likely origin of this outbreak [4,12]. During the decade following this first outbreak, the disease spread throughout the mountain range and into surrounding areas [6]. This epidemiological transition from an initial epizootic outbreak to an endemic disease has been observed in most of the affected Iberian ibex populations [12,13,14,15]. However, the prevalence and mortality rates associated with sarcoptic mange vary between geographic locations and populations [16,17,18].

Given that sarcoptic mange is a concern for both animal health and conservation in the Iberian ibex, several studies have been conducted in order to better understand different aspects of the disease in this species, including ecology, physiology, pathology, genetics, control strategies, and diagnostic methods, among others [14]. However, no large-scale epidemiological studies have been carried out to evaluate the circulation of this parasite in Iberian ibex and sympatric domestic goat populations. The aim of the present study is to assess the exposure and spatial distribution of *S. scabiei* in Iberian ibex and domestic goat populations in Andalusia (southern Spain), the Spanish region with the largest census of both species.

## 2. Materials and Methods

### 2.1. Study Area and Data Collection

During 2015–2021, 411 blood samples from culled Iberian ibexes were collected in the framework of the epidemiological surveillance program coordinated by the Regional Government of Andalusia [19]. Andalusia (southern Spain: 36° N–38°60′ N, 1°75′ W–7°25′ W), the study region, has a predominantly warm Mediterranean climate: mild winters with irregular precipitation, and dry, hot, sunny summers along the coast, becoming more extreme further inland. The average annual temperature is around 18 °C, with over 300 days of sunshine per year. January is the coldest month, while August records the highest temperatures.

**Figure 1:**
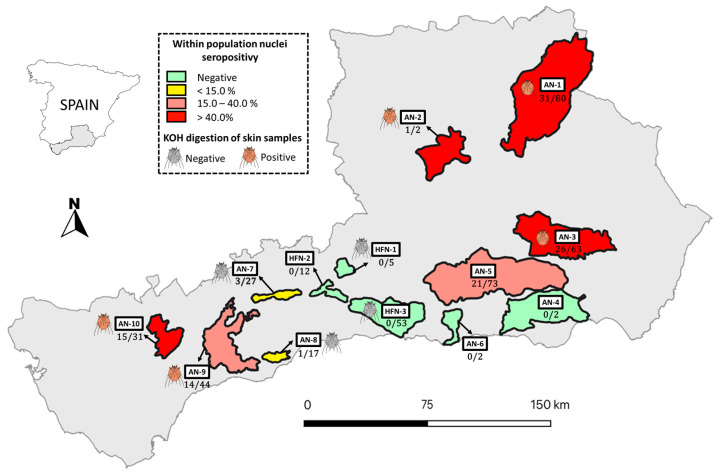
Spatial distribution of the Iberian ibex population nuclei sampled. AN: population nuclei with previous records of sarcoptic mange; HFN: population nuclei historically free of sarcoptic mange. Colour gradation shows within-population nuclei seropositivity. Fractions represent the number of ELISA-positive animals divided by the overall number of animals tested.

Iberian ibexes were opportunistically sampled from the primary population nuclei of this species (*n* = 13), specifically, those with more than one individual per square kilometre. Ten of them were population nuclei with previous records of sarcoptic mange (affected nuclei, AN1-10), while the remaining three population nuclei were historically free of sarcoptic mange (HFN1-3) (Figure 1).

**Figure 2:**
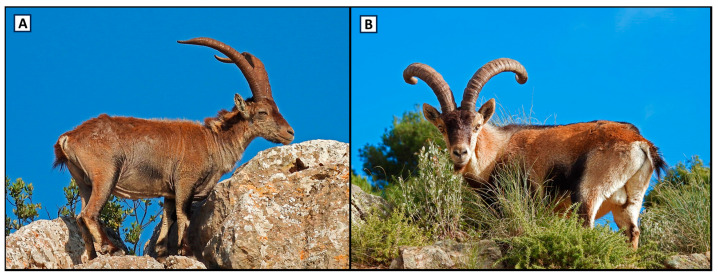
Adult male Iberian ibexes. (**A**) Individual with presence of external mange-compatible lesions. (**B**) Clinically healthy individual.

Sampled animals were classified according to the visual diagnosis of sarcoptic mange and included 157 individuals exhibiting external lesions compatible with sarcoptic mange (alopecia, scales, crusts, seborrhoea, hyperkeratosis, and/or skin lichenification, according to Pérez et al. [20]) and 254 clinically healthy Iberian ibexes (189 from AN and 65 from HFN) (Figure 2). Skin samples were collected from 88 of the 157 ibexes showing mange-compatible lesions. Animals with mange-compatible lesions were divided into four categories according to the percentage of skin surface area affected: grade I (≤25%); grade II, (25–50%); grade III (50–75%); and grade IV (≥75%) [20,21]. Data on location, age (juveniles: <2 years old; sub-adult: 2–6 years old; adult: >6 years old), sex, and date of sampling were recorded in all sampled individuals whenever possible (Table 1).

In addition, cross-sectional sampling was carried out in domestic goats in the same period and study region. The sample size was calculated based on an estimated prevalence of 50% (which provides the highest sample size in studies with unknown prevalence) with a 95% confidence interval (CI95%) and a desired precision of ±5%, resulting in 385 specimens to be sampled. Goat flocks were randomly selected in the four provinces with the highest goat census [22] and included three of the five provinces where Iberian ibexes were sampled. Ultimately, 392 blood samples were randomly collected from 28 goat herds.

### 2.2. Serological Analyses

Blood samples from Iberian ibexes and domestic goats were collected through jugular vein puncture using sterile tubes without anticoagulants in live animals, or by puncture of the endocranial venous sinuses in dead Iberian ibexes, as previously described. After centrifugation, sera were collected and stored at –20 °C until analysis. The presence of antibodies against *S. scabiei* was determined using an in-house indirect ELISA, following the protocol described by Rambozzi et al. [23] but using commercial ELISA plates coated with *S. scabiei* var. *suis* antigen (Sarcoptes-Elisa 2001^®^ PIG, AFOSA GmbH, Blankenfelde-Mahlow, Germany) [24]. The cut-off value used was 92.0% [24]. The estimated sensitivity (Se) and specificity (Sp) of this in-house ELISA were 93.0% and 93.5%, respectively [24].

### 2.3. Potassium Hydroxide (KOH) Digestion Procedure

Skin scrapings were obtained from lesions compatible with mange, encompassing both healthy and injured tissue, and were processed in a 10% KOH solution for 60 min at 37 °C. Subsequently, they were observed under the microscope (20× and 40×) for the detection of *S. scabiei.* Identification of mites was performed according to the keys and descriptions of Wall and Shearer [25].

### 2.4. Statistical Analyses

The frequency of seropositivity was estimated from the ratio of positive samples to the total number of samples analysed. The confidence intervals for seroprevalences were estimated by the standard error 95% confidence interval. Associations between the serological results and independent variables (location, age, sex, sampling year, and percentage of skin surface area affected) were analysed using Pearson’s chi-squared test or Fisher’s exact test, as appropriate. Pearson’s chi-squared test evaluates the observed frequencies from a sample against the expected frequencies, assuming that there is no association between the variables and that the distribution is normal. For the test to be valid, it is essential that all expected frequencies are sufficiently large (>5). Therefore, when there were fewer than six observations per category, Fisher’s exact test was used [26]. Differences were considered statistically significant when *p*-value < 0.05. Statistical analysis to determine significant differences between the serological results and independent variables was carried out using the SPSS statistical software package, version 25.0 (IBM Corporation, Somers, NY, USA).

## 3. Results

Antibodies against *S. scabiei* were detected in 112 (27.3 ± 4.3%) of the 411 Iberian ibexes analysed. Seropositivity was found in 104 of the 157 (66.2 ± 7.4%) animals with mange-compatible lesions, and in 8 of the 254 (3.1 ± 2.2%) clinically healthy ibexes. Out of 88 analysed skin scrapings, mites of *S. scabiei* were confirmed in 57 (64.8%) cases. Antibodies against *S. scabiei* were found in the sera of 49 (86.0 ± 9.0%) of these 57 animals.

At least one seropositive Iberian ibex was detected in 8 of the 13 (61.5%) population nuclei, with seropositive values ranging between 5.8% and 50%. Seropositive animals were detected in AN but not in HFN (Figure 1). Anti-*S. scabiei* antibodies were not detected in any of the 392 domestic goats. Sarcoptic mange-compatible lesions were not observed by farmers in the 28 goat flocks sampled.

Significant differences in seropositivity were observed among Iberian ibexes with mange-compatible lesions, depending on the affected skin surface. The frequency of antibodies significantly increased with the percentage of skin surface affected, as follows: 51.1 ± 14.6% (23/45) with grade I; 76.3 ± 13.5% (29/38) with grade II; 83.3 ± 21.1% (10/12) with grade III; and 88.2 ± 10.8% (30/34) with grade IV (*p* = 0.002) (Table 1). A temporal pattern was also observed, with a significantly higher seropositivity in spring (42.9 ± 8.2%; 60/140) compared to summer (23.7 ± 13.5%; 9/38; *p* = 0.023), autumn (8.8 ± 5.2%; 10/114; *p* < 0.001), and winter (27.7 ± 8.0%; 33/119; *p* = 0.008).

## 4. Discussion

Even though different direct and indirect methods have been proposed to investigate the circulation of *S. scabiei* in wild and domestic species—including clinical diagnosis, dermatoscopy, intradermal skin tests, infrared thermal imaging, antibody and antigen detection, PCR-based methods, or even the use of mange-detector dogs [5,27,28,29]—the diagnosis of sarcoptic mange remains a challenge today. Currently, there are few diagnostic methods showing satisfactory performance and cost–benefit balance [30]. Among them, indirect ELISA is deemed a valuable diagnostic tool for assessing *S. scabiei* exposure [2,24,30].

The overall individual prevalence of antibodies against *S. scabiei* detected in the Iberian ibex (27.3%) during the present study is evidence of a high ongoing circulation of the parasite in southern Spain. However, given that the analysed animals were opportunistically sampled in the framework of the epidemiological surveillance program, this result may eventually overestimate the actual prevalence of sarcoptic mange among them, thus, the outcome should be interpreted taking into account the different epidemiological contexts. Moreover, these results should be interpreted in the context of a broad time frame, taking into account that the risk factors could change over the time. Of the Iberian ibexes exhibiting mange-compatible lesions, 66.2% tested positive for anti-*S. scabiei* antibodies. This result is mostly explained by the lower Sp (60.7%) of the visual diagnosis of sarcoptic mange in Iberian ibexes [31] compared to the ELISA (93.5%) [24]. This interpretation is supported by the higher seroprevalence (86.0%) found in ibexes that exhibited mites of *S. scabiei* in their skin samples. However, eight cases of mange confirmed by KOH digestion were seronegative, confirming the estimated Se value of the ELISA test used. Complementarily, the onset of clinical dermatological signs following *S. scabiei* infestation may shortly anticipate the onset of a measurable humoral response, as shown by experimental trials in the Iberian ibex [24,32] and in other mammal species [33,34,35,36]. Consistently, within the group of animals showing mange-compatible lesions, we found a significant positive association between the presence of anti-*S. scabiei* antibodies and the affected skin surface—a reasonable proxy of the mite population size and the related antigenic stimulation of the host’s immune system [20]. These findings suggest a cumulative persistence of antibodies throughout the course of the disease; moreover, they are further evidence of the poor defensive contribution of the circulating antibody response in the face of *Sarcoptes* invasion [2,18,37].

Interestingly, 3.1% of the clinically healthy Iberian ibexes showed antibodies against *S. scabiei.* This result could be associated to the Sp of the ELISA (93.5%) or may mirror alternative situations, such as (i) recovery after infection and the transitory development of mild lesions [15,38,39], or (ii) the presence of the disease at an early stage. Concerning the second hypothesis, although the Se of the visual inspection has been shown to be high (87.1%) [31], the presence of undetected skin lesions during the external inspection cannot be ruled out, particularly in recently infected animals. Additional studies are needed to evaluate these hypotheses.

A significantly higher seropositivity was observed in animals sampled in spring. This result is consistent with the higher number of cases of sarcoptic mange reported in Iberian ibexes during the winter season [12,27,31,40], as well as the subsequent seroconversion several weeks after infestation [32]. The cold period is particularly favourable for mite direct transmission because of the timing of the rutting season [11]. This temporal pattern can be also explained by the highest survival time of *S. scabiei* at <20 °C and relative humidity > 75% [2], which increases the risk of environmental (indirect) transmission during the winter period.

The detection of at least one seropositive animal in eight of the thirteen (61.5%) sampled population nuclei indicates that *S. scabiei* is widespread in this species in southern Spain. Accordingly, Fernández-Muñoz et al. [14] observed that sarcoptic mange is rapidly spreading among Iberian ibex populations across the Iberian Peninsula, particularly in the Mediterranean Basin. In our study, all seropositive individuals originated from AN, whereas seropositivity was not found in the Iberian ibexes from the three HFN analysed. Of note, the number of analysed sera from the remaining two nuclei was very limited (two samples each, Figure 1). From a conservation perspective, we highlight an interest in confirming, with a diagnostic method complementary to the traditional ones (e.g., observation from a distance), that no circulation of the parasite occurred in selected areas during the study period, despite proximity and likely connections with AN. The reasons why these nuclei have been mange-free for decades are unknown and undoubtedly worth investigating. Resilience in naïve nuclei of Iberian ibex and other caprine hosts to the severe demographic effects of sarcoptic mange has been already reported [8,15,41] but an authentic innate resistance would be unprecedented. If they exist, innately resistant nuclei should represent a favourite source of founder individuals for (i) planned reintroductions in areas connected with AN, and (ii) the restocking of those ibex nuclei which are still very sensitive to mange effects after decades of *Sarcoptes* circulation.

Since the first epizootic outbreak of sarcoptic mange was described in Iberian ibex populations in late 1987 [12], different studies have pointed out the relevance of multi-host systems in the transmission and maintenance of *S. scabiei* [4]. In this sense, transmission of *S. scabiei* at the wild Caprinae–domestic goat interface has been well documented [7,12,42,43]. In our study, none of the domestic goats analysed showed antibodies against this parasite, suggesting that sarcoptic mange is now maintained in an independent wild cycle in the study area. In line with this, Falconi et al. [44] observed that *S. scabiei* is self-maintained independently in Cantabrian chamois (*Rupicapra pyrenaica parva*) and livestock in northern Spain.

## 5. Conclusions

To the best of the authors’ knowledge, this is the first seroepidemiological study that jointly assesses the exposure of *S. scabiei* at the Iberian ibex–domestic goat interface. Our results indicate a widespread distribution of this parasite among the Iberian Ibex populations of southern Spain and a null or limited circulation in domestic goat flocks from this region, suggesting that *S. scabiei* is maintained in exposed ibex nuclei, and in sympatric livestock if justified by the epidemiological context. Surveillance should inform the management of exposed ibex nuclei, including options such as selective culling of mangy individuals, depopulation, mass treatment, and *laissez-faire* [4,6]. Within this frame, the results of our survey suggest that sero-diagnosis by indirect ELISA represents a useful complementary tool to monitor the exposure of wild and domestic Caprinae to *S. scabiei* over time.

## Figures and Tables

**Table 1:** Frequency of antibodies against *S. scabiei* in Iberian ibexes in Andalusia (southern Spain) and results of bivariate analysis.

Variable	Categories	Iberian Ibexes with Skin Lesions Compatible with Sarcoptic Mange			Clinically Healthy Iberian Ibexes		
		% ELISA Positive	Seropositives/ Overall ^a^	*p*-Value	% ELISA Positive	Seropositives/ Overall ^a^	*p*-Value
Location ^b^	AN HFN	68.4 0.0	104/152 0/5	0.004	4.2 0.0	8/189 0/65	0.09
Age	Juvenile Sub-adult Adult	100.0 67.1 63.9	7/7 49/73 46/72	0.152	10.0 4.5 2.1	1/10 4/88 3/140	0.305
Sex	Male Female	68.1 58.5	77/113 24/41	0.179	3.7 1.6	7/187 1/62	0.366
Sampling year	2015 2016 2017 2018 2019 2020 2021	- 0.0 60.6 73.3 66.1 80.0 68.8	- 0/3 20/33 11/15 39/59 12/15 22/32	0.153	20.0 2.8 1.7 6.8 1.5 3.4 0.0	1/5 1/36 1/58 3/44 1/66 1/29 0/16	0.231
Percentage of skin surface area affected	Grade I (≤25%) Grade II (25–50%) Grade III (50–75%) Grade IV (≥75%)	51.1 76.3 83.3 88.2	23/45 29/38 10/12 30/34	0.002	-	-	-

^a^ Missing values omitted. ^b^ AN: population nuclei with previous records of sarcoptic mange; HFN: population nuclei historically free of sarcoptic mange.

## Data Availability

The data presented in this study are available on request from the corresponding author.
